# University common spaces and students’ psychological restoration: a scoping review of environmental characteristics, restorative outcomes, and potential pathways

**DOI:** 10.3389/fpubh.2026.1955565

**Published:** 2026-10-28

**Authors:** Hongyan Wen, Hanzheng Lin

**Affiliations:** 1 Faculty of Tourism & Cultural Creativity, Fujian Polytechnic of Information Technology, Fuzhou, China; 2 College of Design and Creativity, Fujian Jiangxia University, Fuzhou, China

**Keywords:** restorative environments, university common spaces, psychological restoration, influencing factors, pathways of influence

## Abstract

**Background:**

University common spaces are important settings where university students spend time, move between destinations, relax, and interact socially. Their environmental characteristics may influence attention restoration, stress reduction, and emotion regulation.

**Methods:**

Following the PRISMA-ScR reporting guidelines for scoping reviews, we systematically searched the relevant literature, and conducted a narrative synthesis of 52 studies that met the inclusion criteria. The synthesis characterized the current state of research on how university common spaces promote psychological restoration among university students, including the influencing factors, psychologically restorative outcomes, and potential pathways underlying these effects.

**Results:**

The results showed the following: (1) Existing studies have focused primarily on campus landscape green spaces, particularly vegetated spaces and waterfront spaces, whereas architectural-environmental and facility-support factors have received relatively little attention. (2) University students’ psychological restoration has mainly been assessed using five categories of indicators: attention restoration, stress reduction, regulation of negative emotions, vitality restoration, and reduced loneliness. All five indicators are influenced, to varying degrees, by the characteristics of university common spaces and their constituent elements. (3) The characteristics and constituent elements of university common spaces may influence university students’ psychological restoration both directly and indirectly, with the latter effects mediated by attitudinal perceptions and behavioral support, including perceived restorativeness, environmental satisfaction, spatial preference, place attachment, physical activity, and social interaction.

**Conclusion:**

Overall, university common spaces have the potential to promote psychological restoration among university students; however, existing studies remain limited by insufficient coverage of space types, a lack of systematic measurement of constituent elements, a lack of standardized restoration outcomes, and limited evidence on underlying mechanisms. Future research should compare multiple types of university common spaces, integrate outcome measures, identify pathways, and quantify spatial elements to provide more actionable evidence for designing restorative environments on university campuses.

## 1 Introduction

University students are at a critical developmental stage in which academic development, social adaptation, and self-construction intersect. Sustained academic pressure, attentional fatigue, and the accumulation of negative emotions have prompted increasing concern about their mental health (1, 2). University common spaces provide important settings where students spend time, move between destinations, relax, and interact socially in their daily lives. In addition to organizing circulation and enhancing the campus landscape, these spaces may support psychological restoration by providing opportunities for relaxation, psychological distance from everyday stressors, and contact with nature.

Research on restorative environments provides an important theoretical basis for understanding the relationship between spatial environments and psychological restoration. Attention Restoration Theory proposes that environments characterized by being away, fascination, extent, and compatibility can help alleviate directed-attention fatigue (3). Stress Reduction Theory, in turn, suggests that environments with positively perceived characteristics can reduce stress responses and promote relaxation (4). As research has expanded from natural settings to urban and built environments, restorative-environment theory has increasingly been applied in architecture, landscape architecture, urban and rural planning, and related fields to explain how specific spatial designs influence users’ psychological states (5).

Within university settings, research has increasingly examined the restorative potential of university common spaces. However, much of this literature has focused on campus green spaces and landscape features, particularly vegetation and water, which have been associated with positive emotions, stress reduction, and attention restoration (6, 7). The existing evidence remains uneven in both research scope and methodology. Most studies have concentrated on campus landscape green spaces, with relatively limited attention given to architectural-environmental and facility-support features (8–11). Moreover, studies have primarily examined the direct effects of spatial characteristics on restoration outcomes, whereas relatively few have explored mediating pathways involving environmental attitudes, physical activity, and social interaction (12–16).

In this review, university common spaces refer to shared outdoor settings on university campuses that students can access and use for everyday activities, including rest, movement, learning, social interaction, and physical activity. Vegetated spaces, waterfront spaces, plazas, courtyards, sports fields, pedestrian paths, and therapeutic gardens are grouped under this concept because they provide shared settings for students’ everyday environmental exposure and activities, despite differences in their physical form and primary function. This grouping does not imply that these spaces have equivalent restorative qualities or operate through identical pathways. University common spaces encompass natural, built, and activity-supportive features. Their potential to promote psychological restoration is therefore unlikely to depend solely on landscape elements such as vegetation or water. Plazas, courtyards, pedestrian paths, sports fields, therapeutic gardens, and various types of campus green spaces may shape students’ perceptual experiences and behavioral choices through characteristics such as spatial openness, perceived naturalness, facility usability, accessibility, and activity support (17–20). Accordingly, evaluating university common spaces solely in terms of the extent of greening or a single landscape element may not fully explain their psychologically restorative potential.

Psychological restoration among university students is also a multidimensional construct. It includes attention restoration, stress reduction, and emotion regulation, but may also involve increased vitality, reduced loneliness, improved psychological well-being, and enhanced quality of life (8, 21–24). Existing studies often operationalize psychological restoration using a single scale or indicator. Although this approach facilitates the examination of specific outcomes, it may obscure the relationships among different dimensions of restoration (13, 25, 26). Given the substantial heterogeneity in research perspectives, spatial types, indicator selection, and measurement tools, a systematic synthesis of the psychologically restorative effects of university common spaces remains lacking.

Against this background, we conducted a scoping review in accordance with the PRISMA-ScR reporting guidelines to systematically synthesize research on university common spaces and university students’ psychological restoration. Specifically, this review addressed three questions: First, which environmental characteristics and constituent elements of university common spaces have been examined in relation to university students’ psychological restoration? Second, which indicators and measurement tools have most commonly been used to assess students’ psychologically restorative outcomes? Third, through which direct or indirect pathways might the characteristics of university common spaces influence students’ psychological restoration? By clarifying the scope, areas of consensus, and limitations of the existing evidence, this study aims to inform future research and provide a theoretical basis to inform healthy-campus policy and design practice.

## 2 Materials and methods

### 2.1 Search strategy

We conducted a scoping review to collect and appraise the literature, identify knowledge gaps (27), and determine the factors influencing university students’ psychological restoration in accordance with the research questions. Following the PRISMA Extension for Scoping Reviews (PRISMA-ScR) (28), the review process comprised four stages: identification, screening, eligibility assessment, and inclusion. We searched six databases—Web of Science Core Collection, MEDLINE, PubMed, PsycArticles, GreenFILE, and China National Knowledge Infrastructure (CNKI)—for literature published before October 2024, using the first online publication date of the version of record as the reference point. The CNKI search was conducted using Chinese-language terms. English translations are presented in Table 1 for readability; these translations were not used to execute the CNKI search.

**Table 1:** Concept blocks and English-language search terms used in the literature search.

Concept	Search terms
Population	“university student*” OR “college student*” OR “student*”
Spatial setting	“university campus common space*” OR “university campus public space*” OR “university campus outdoor space*” OR “university campus open space*” OR “university campus environment*”
Outcomes	“psychological restoration” OR “psychological recovery” OR “psychological* restorative effect*” OR “psychological health” OR “mental health” OR “psychological well-being” OR “mental well-being” OR “psychological benefit*” OR “psychological support” OR “psychological adjustment” OR “attention restoration” OR “stress reduction”

The asterisk (*) is a wildcard representing any character string and was used to retrieve words with different endings. CNKI was searched using Chinese-language terms. English equivalents are shown here for reference.

The search strategy was developed in accordance with the PECO framework, and the search terms were organized into three conceptual blocks: population, spatial setting, and psychologically restorative outcomes (29). Within each block, terms were combined using “OR,” whereas the three blocks were combined using “AND.” The population block included “university student*” OR “college student*” OR “student*.” The spatial-setting block included “university campus common space*” OR “university campus public space*” OR “university campus outdoor space*” OR “university campus open space*” OR “university campus environment*.” The outcome block included “psychological restoration” OR “psychological recovery” OR “psychological* restorative effect*” OR “psychological health” OR “mental health” OR “psychological well-being” OR “mental well-being” OR “psychological benefit*” OR “psychological support” OR “psychological adjustment” OR “attention restoration” OR “stress reduction.”

Because measures of psychological restoration do not always use the term “restoration” directly, but may instead refer to health, well-being, benefits, support, or adjustment, the outcome block incorporated related terms covering psychological health, psychological well-being, psychological benefits, psychological support, psychological adjustment, attention restoration, and stress reduction. Using these search terms (Table 1), we searched six databases using subject-heading, title, abstract, and keyword fields. Because the included studies comprised quantitative, qualitative, and mixed-methods research, and some studies did not include an explicit comparator group, the comparator component was not imposed as a mandatory search block. Instead, the relevance of the comparator was assessed during the screening process according to the study design and research question.

### 2.2 Screening criteria

All retrieved records were first imported into Zotero for reference management and deduplication. Two reviewers then independently screened the titles and abstracts against the prespecified inclusion and exclusion criteria. Any record considered potentially eligible by either reviewer proceeded to full-text assessment to minimize the risk of missing relevant studies. The two reviewers independently assessed the full texts according to the following inclusion criteria: (1) publication in a peer-reviewed journal; (2) inclusion of university students as participants; (3) investigation conducted in university common spaces, such as vegetated areas, waterfronts, plazas, courtyards, or sports fields; (4) examination of the relationship between university common space characteristics and students’ psychological restoration or related mental health outcomes; (5) use of quantitative, qualitative, or mixed methods in an empirical study.

Any disagreements arising during title and abstract screening or full-text assessment were resolved by jointly re-examining the relevant article and discussing it with reference to the prespecified eligibility criteria until consensus was reached. Following the initial database search, the reference lists of relevant reviews and included studies were manually screened to identify additional articles that may have been missed by the database searches. These procedures resulted in the inclusion of 52 studies were ultimately included. The study identification, screening, and inclusion process is summarized in Figure 1.

**Figure 1:**
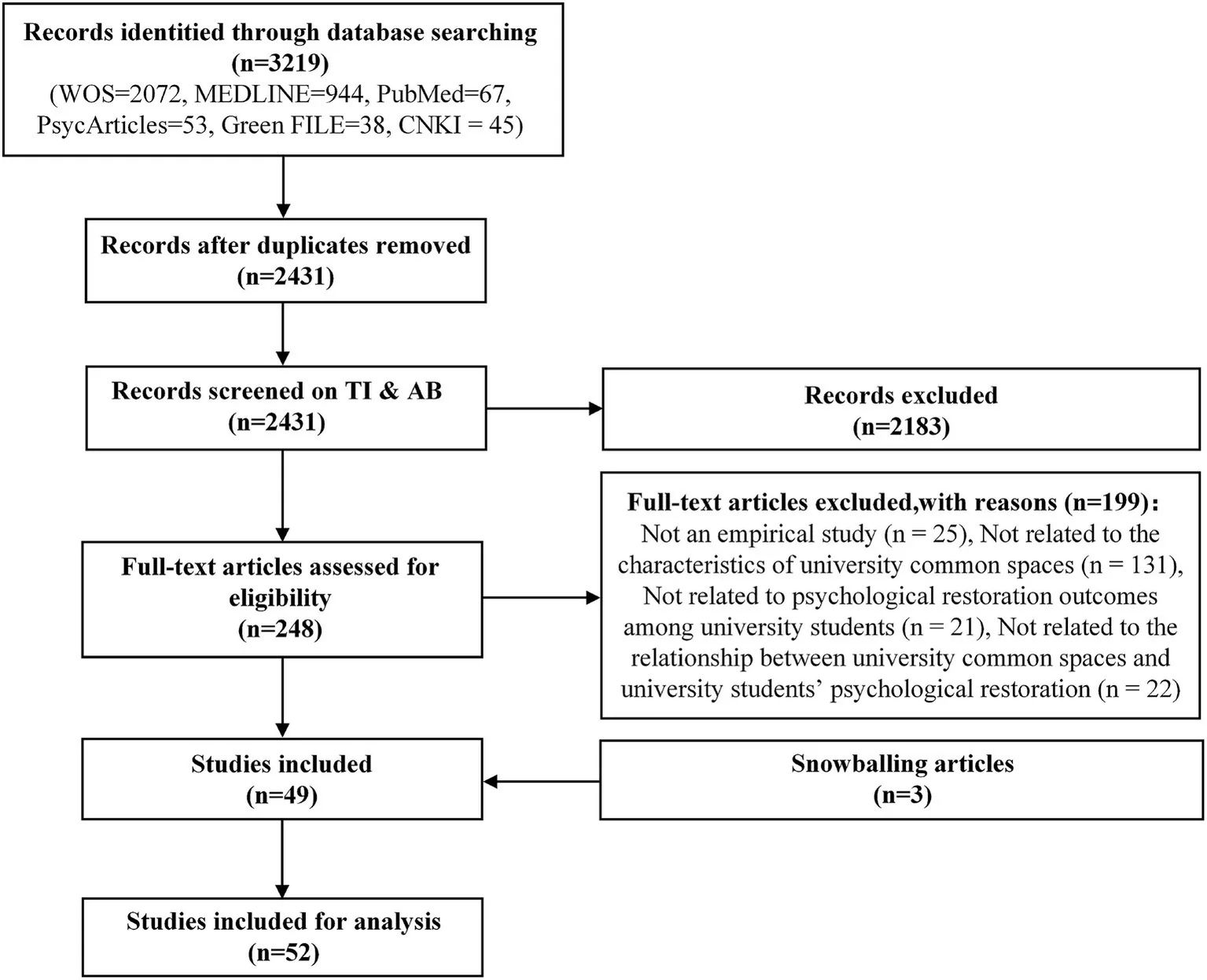
PRISMA flow diagram of the study selection process.

### 2.3 Data extraction

We used a prespecified data-extraction form was used to extract information from the included studies. Data extraction was conducted independently by two reviewers, after which the extracted information was cross-checked item by item. Any discrepancies were resolved by re-examining the original article and reaching consensus through discussion. The extracted information covered four main domains: (1) background characteristics, including study title, authors, journal, publication year, and study location; (2) methodological characteristics, including study population, sample size, study design, data-collection methods, and measurement instruments; (3) study variables, including university common space characteristics, psychologically restorative outcomes, and relevant demographic characteristics; and (4) key findings, including the relationships between spatial characteristics and psychologically restorative outcomes, the direction of these relationships, and the principal conclusions reported by the authors. The extracted data were subsequently coded, categorized, and compared to identify emerging trends, commonalities, and differences across studies. Given the relatively small number of included studies and the substantial heterogeneity in study designs, measurement instruments, and outcome reporting, a narrative synthesis was conducted to summarize the relationship between university common spaces and students’ psychological restoration.

## 3 Results

### 3.1 Characteristics of the existing literature

The initial search identified 3,219 relevant records (WOS = 2,072; MEDLINE = 944; PubMed = 67; PsycArticles = 53; GreenFILE = 38; CNKI = 45). After 788 duplicate records were removed, 2,431 records remained. Based on their titles and abstracts, 248 records were selected for full-text screening. Of these, 199 were excluded because 25 were not empirical studies, 131 did not examine characteristics of university common spaces, 21 did not assess university students’ psychologically restorative outcomes, and 22 did not analyze the relationship between the characteristics of university common spaces and students’ psychological restoration. Three additional relevant studies were identified from the reference lists of previous reviews, bringing the total to 52 studies being included in the final analysis (Figure 1, Table 2).

**Table 2:** Studies included in this review (*n* = 52).

Study ID	References	Participants	Sample size	Study design	Data collection	Common space types	Spatial elements	Psychologically restorative outcomes	Pathways of influence	Relationship model
1	Hipp et al. (24)	university students	*n* = 441	quantitative	questionnaire survey	campus green spaces	perceived naturalness	quality of life	indirect effect via perceived restorativeness	+
2	Liprini et al. (38)	full-time students	*n* = 286	quantitative	questionnaire survey	campus green spaces	tree species; plant species; tree–shrub composition	attention restoration	direct effect	+
3	Bogerd et al. (43)	students	*n* = 722	quantitative	questionnaire survey	campus green spaces	green-space area; rest facilities; art installations	vitality restoration	direct effect	+
4	Ibes et al. (34)	undergraduate and postgraduate students	*n* = 558	qualitative	interviews	campus green spaces	tranquil atmosphere; perceived naturalness; accessibility	stress reduction	direct effect	+
5	Wang et al. (26)	university students	*n* = 323	quantitative	questionnaire survey	campus landscapes	water features; vegetation coverage	stress reduction	direct effect	+
6	Holt et al. (50)	undergraduates	*n* = 207	quantitative	questionnaire survey	campus green spaces	accessible paths; natural elements; water features	psychological well-being	direct effect	±
7	Gulwadi et al. (55)	undergraduates	*n* = 1,079	quantitative	questionnaire survey	campus green spaces	green-space coverage; perceived naturalnes	quality of life	indirect effect via perceived restorativeness	+
8	Lu and Fu (32)	university students	*n* = 360	quantitative	questionnaire survey	common spaces (waterfront, vegetation, courtyards, plazas)	spatial visibility; spatial accessibility; spatial comfort; spatial recognition; sense of spatial belonging	attention restoration	direct effect	+
9	Malekinezhad et al. (8)	university students	*n* = 444	quantitative	questionnaire survey	campus green spaces	green-space quality	attention restoration; reduced loneliness; vitality restoration	indirect effect via perceived restorativeness	+
10	Ibes and Forestell (9)	university students	*n* = 234	quasi-experimental (pretest–posttest)	questionnaire survey	campus green spaces	green-space accessibility; perceived naturalness	regulation of negative emotions	direct effect	+
11	Guo et al. (20)	postgraduates	*n* = 150	natural experiment	questionnaire survey	pedestrian paths	tree color; canopy size; trunk form	stress reduction	direct effect	+
12	Foellmer et al. (57)	undergraduate and postgraduate students	*n* = 100	mixed methods (qualitative and quantitative)	questionnaire survey; interviews	campus green spaces	green-space area; green-space location	psychological well-being	direct effect	±
13	Shrestha et al. (10)	university students	*n* = 13	mixed methods (qualitative and quantitative)	questionnaire survey; interviews	campus green spaces	perceived naturalness	regulation of negative emotions	direct effect	+
14	Sun et al. (37)	university students	*n* = 819	quantitative	questionnaire survey	common spaces (waterfront, vegetation, grey spaces, sports fields)	plant diversity; water features; activity facilities	attention restoration; stress reduction; regulation of negative emotions	direct effect	+
15	Ha et al. (6)	university students	*n* = 319	quasi-experimental (pretest–posttest)	questionnaire survey	campus landscapes	biodiversity; natural soundscape	regulation of negative emotions	direct effect	+
16	Stepansky et al. (11)	students aged over 18	*n* = 62	mixed methods (qualitative and quantitative)	questionnaire survey; interviews	campus green spaces	diverse trees and plants; sufficient rest facilities; activity areas	regulation of negative emotions	direct effect	+
17	Huang and Cao (30)	from different disciplines students	*n* = 12	psychological experiment	questionnaire survey	courtyard spaces	scale of building enclosures; building height; transparency of building interfaces; sky visibility	environmental satisfaction	direct effect	±
18	Peng et al. (31)	with educational backgrounds in architecture and urban–rural planning university students	*n* = 139	virtual-simulation experiment	questionnaire survey	campus green spaces	green-space diversity; sky visibility	regulation of negative emotions	direct effect	±
19	Zhang et al. (47)	university students	*n* = 30	quasi-experimental (pretest–posttest)	questionnaire survey	campus green spaces	plant species; water-feature quality	regulation of negative emotions	direct effect	+
20	Liu et al. (35)	university students	*n* = 474	quantitative	questionnaire survey	campus green spaces	perceived naturalness	regulation of negative emotions	indirect effect via place attachment and landscape preference	+
21	Boyd (12)	aged 18–24 undergraduates	*n* = 55	quantitative	questionnaire survey	campus green spaces	mature trees; flowering plants; naturalistic planting form	mental health	direct effect	+
22	Du et al. (7)	undergraduates	*n* = 243	quantitative	questionnaire survey	common spaces (waterfront, vegetation, commuting paths, sports fields)	perceived naturalness	attention restoration; stress reduction	direct effect	+
23	Liu et al. (56)	university students	*n* = 1,236	quantitative	questionnaire survey	campus green spaces	green-space layout; plant diversity;	mental health	indirect effect via learning engagement	+
24	Lee et al. (48)	university students	*n* = 788	quantitative	questionnaire survey	waterfront spaces	water features; plant diversity	stress reduction; regulation of negative emotions	direct effect	+
25	DeLauer et al. (13)	university students	*n* = 71	qualitative	interviews	campus landscapes	plant characteristics; art installations	regulation of negative emotions	direct effect	+
26	Liu et al. (56)	students	*n* = 897	quantitative	questionnaire survey	campus green spaces	perceived naturalness	attention restoration; regulation of negative emotions	indirect effect via self-rated restoration	+
27	Kiers et al. (25)	university students	*n* = 265	quantitative	questionnaire survey	campus green spaces	biodiversity	regulation of negative emotions	direct effect	+
28	Guo et al. (17)	university students	*n* = 478	quantitative	questionnaire survey	common spaces (vegetation, waterfront, plazas, courtyards, sports fields)	architectural-environmental characteristics; landscape-environmental characteristics; rest-facility characteristics; activity-facility characteristics;	attention restoration; stress reduction	indirect effect via static restorative and dynamic exercise behaviors	+
29	Asim et al. (41)	aged 18–23 university students	Sample 1 (*n* = 429); Sample 2 (*n* = 22)	mixed methods (qualitative and quantitative)	questionnaire survey	campus landscapes	building form; green vegetation; open views	regulation of negative emotions	direct effect	±
30	Migl et al. (14)	undergraduates	*n* = 122	qualitative	questionnaire survey	campus green spaces	perceived naturalness	regulation of negative emotions	direct effect	+
31	Ning et al. (15)	second-year landscape-architecture students	*n* = 60	natural experiment	questionnaire survey	campus green spaces	plant species; water-feature quality	regulation of negative emotions; stress reduction	direct effect	+
32	Sun et al. (44)	university students	*n* = 1,236	quantitative	questionnaire survey	campus green spaces	green-space layout; plant diversity;	mental health	indirect effect via learning engagement	+
33	Meng and Xu (58)	university students	*n* = 188	qualitative	interviews	campus landscapes	tranquil atmosphere; water features; biodiversity	regulation of negative emotions	direct effect	+
34	Ma et al. (39)	students	*n* = 123 (aged 20–24)	quantitative	questionnaire survey	campus landscapes	water features; tree forms; plant colors	attention restoration	direct effect	+
35	Kanelli et al. (16)	university students	*n* = 108	quantitative	questionnaire survey	campus green spaces	green-space availability; perceived naturalness; spatial safety; spatial quality (aesthetics, attractiveness and biodiversity)	attention restoration	direct effect	+
36	Delbert et al. (19)	undergraduate and postgraduate students	*n* = 12	qualitative	interviews	therapeutic gardens	spatial location; diverse plants; visual focal points; tranquil atmosphere	stress reduction	direct effect	+
37	Xu et al. (36)	undergraduate and postgraduate students	*n* = 31	natural experiment	questionnaire survey	campus green spaces	pleasant soundscape; vegetation visibility; sky visibility	regulation of negative emotions	indirect effect via perceived restorativeness	+
38	Zhou et al. (22)	students	*n* = 346	quantitative	questionnaire survey	campus green spaces	green-space availability; green-space attractiveness; green-space visibility	psychological well-being	indirect effect via green-space satisfaction and social-environmental satisfaction	+
39	Zhang et al. (45)	undergraduate and postgraduate students	*n* = 90	natural experiment	questionnaire survey	campus landscapes	water features; plant color; spatial location; facility maintenance	regulation of negative emotions	direct effect	+
40	Huang et al. (40)	students	*n* = 537	quantitative	questionnaire survey	pedestrian paths	building height; tree height; shrub area	attention restoration	direct effect	+
41	Mao et al. (33)	university students	*n* = 820	quantitative	questionnaire survey	campus green spaces	green-space visibility; green-space area	mental health	indirect effect via physical activity and social cohesion	±
42	Sun et al. (42)	aged 18–26 university students	*n* = 41	natural experiment	questionnaire survey	campus landscapes	visual complexity; perceived naturalness; environmental color	stress reduction	direct effect	+
43	Zhang et al. (45)	university students	*n* = 550	quantitative	questionnaire survey	campus green spaces	tranquil atmosphere	attention restoration	indirect effect via perceived restorativeness and solitude competence	+
44	Bai et al. (23)	university students	*n* = 811	quantitative	questionnaire survey	campus green spaces	green-space area	psychological well-being	direct effect	+
45	Zheng et al. (66)	university students	*n* = 1,173	quantitative	questionnaire survey	campus landscapes	sky visibility; spatial openness	mental health	indirect effect via sports participation	+
46	Nie et al. (61)	university students	*n* = 2,528	quantitative	questionnaire survey	campus landscapes	seating elements; canopy size; plant elements; sidewalks and paths;	psychological well-being	direct effect	+
47	Wen et al. (18)	university students	*n* = 575	quantitative	questionnaire survey	common spaces (vegetation, waterfront, plazas, courtyards, sports fields)	green view ratio; water-feature coverage; openness of building enclosures	attention restoration; stress reduction	direct effect	+
48	Liu and Li et al. (54)	university students	*n* = 609	quantitative	questionnaire survey	campus green spaces	green-space perimeter-to- area ratio; green-space visibility; green-space accessibility; green-space ratio	regulation of negative emotions	indirect effect via green-space visitation behavior	+
49	Wang and Mei (46)	students	*n* = 20	qualitative	interviews	campus common spaces	spatial forms; accessibility; activity support; natural characteristics; perceptual experience	attention restoration	direct effect	+
50	Zhou et al. (49)	undergraduate and postgraduate students	*n* = 62	virtual-simulation experiment	questionnaire survey	campus landscapes	visual elements (lawns, water bodies, woodlands, plazas); auditory elements (natural sounds, everyday sounds, mechanical sounds, silence)	regulation of negative emotions	direct effect	+
51	Zhou et al. (53)	students	*n* = 90	natural experiment	questionnaire survey	campus landscapes	perceived greenery; sense of nature; coherence; comfort; site attractiveness; sense of artistic conception; atmosphere; interestingness	stress reduction; regulation of negative emotions	direct effect	+
52	Weng et al. (51)	university students	*n* = 30	natural experiment	questionnaire survey	campus green spaces	natural sounds; traffic sounds; anthropogenic sounds	attention restoration; regulation of negative emotions	direct effect	+

Study ID 47 (18) was first published online as the Version of Record in September 2024 and was subsequently assigned to Volume 110 (2025). For this review, it was classified as a 2024 publication based on its first online publication date.

Based on the first online publication date of the version of record, all selected studies were published between 2016 and 2024, and the number of publications increased markedly after 2021, with studies published in 2024 accounting for the largest share (31%) of the selected literature (Figure 2). Most studies were conducted in Asia, North America, or Europe; approximately 90% of the included articles were published in journals based outside China, whereas 10% were published in China-based journals. More than half of the 52 studies were published in the International Journal of Environmental Research and Public Health (*n* = 9), Frontiers in Public Health (*n* = 5), Urban Forestry & Urban Greening (*n* = 5), Building and Environment (*n* = 3), BMC Public Health (*n* = 2), and Chinese Landscape Architecture (*n* = 2) (Figure 3).

**Figure 2:**
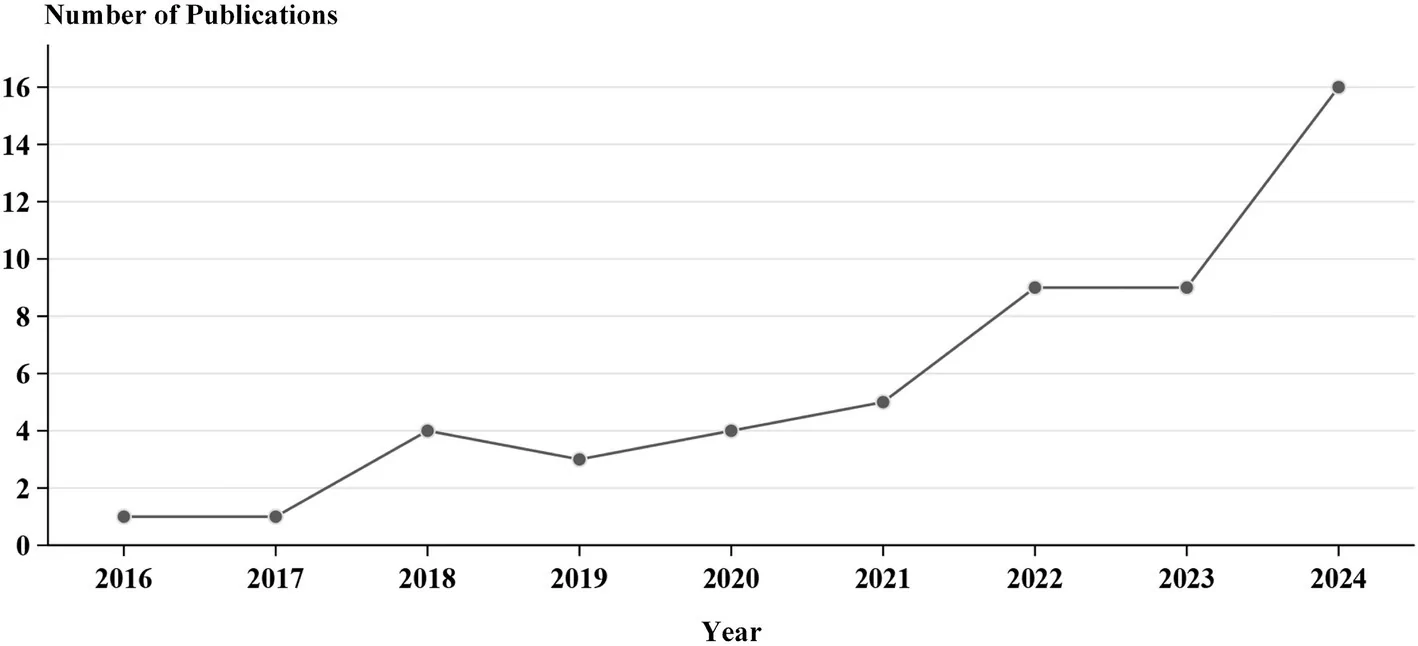
Annual trends in the number of publications.

**Figure 3:**
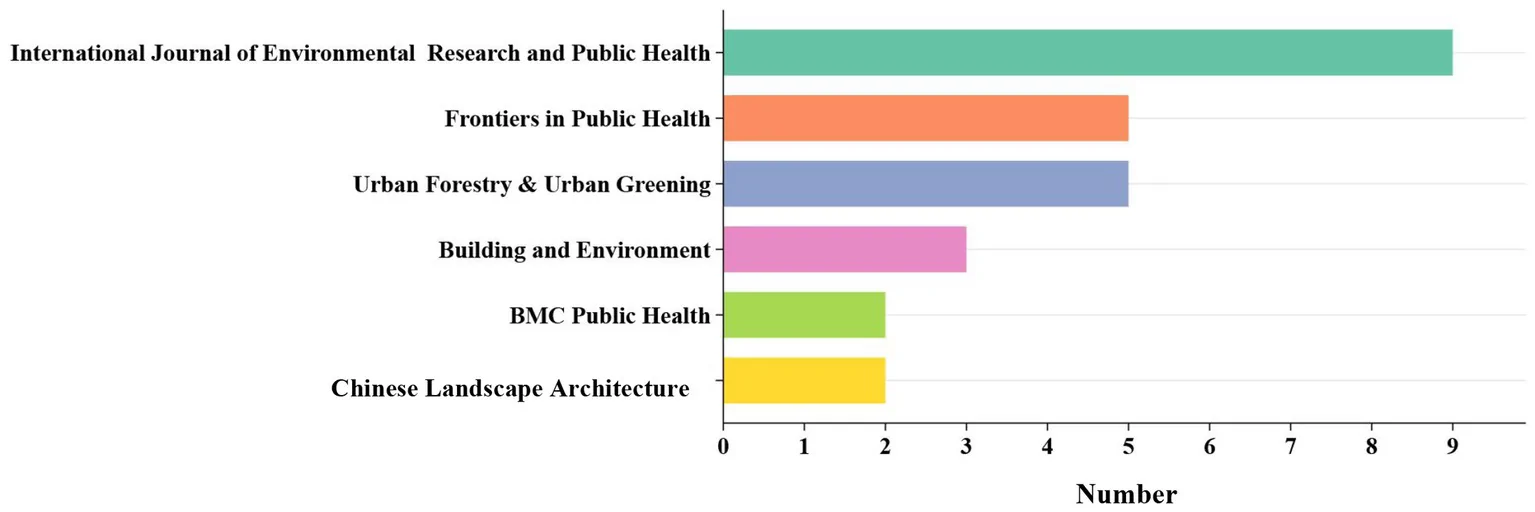
Ranking of journals by number of publications.

Among the 52 included studies, 29 were cross-sectional quantitative studies, 13 were quasi-experimental studies, 6 were qualitative studies, and 4 used mixed methods (Figure 4; Table 2). The four mixed-methods studies combined qualitative and quantitative approaches. The 13 quasi-experimental studies comprised one psychological experiment (30), two virtual-simulation experiments (31), three pilot studies using pretest–posttest designs, and seven natural experiments. Questionnaires were widely used in quantitative and experimental studies, interviews were the principal method used in qualitative studies, and mixed-methods studies combined interviews with questionnaires.

**Figure 4:**
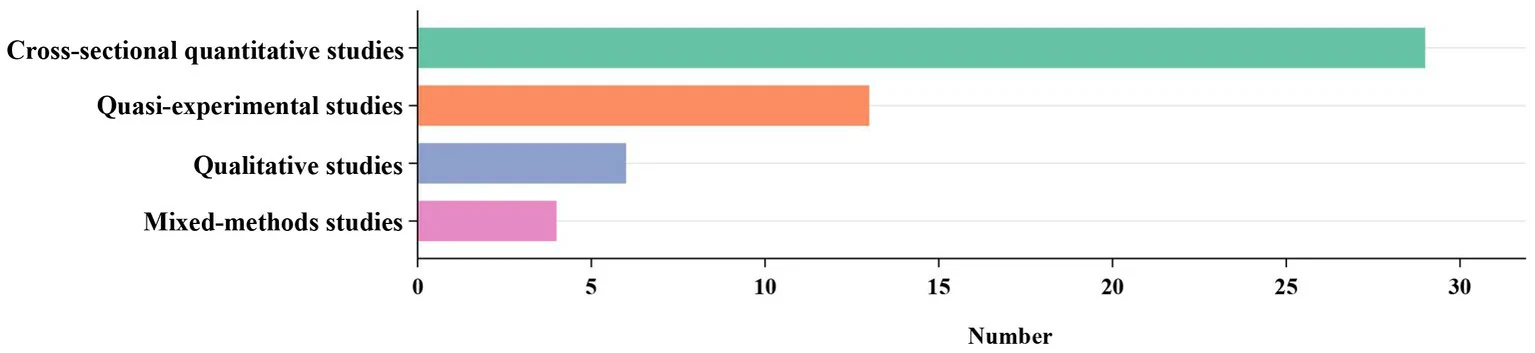
Distribution of research designs used in the existing literature.

Most studies focused on campus landscape green spaces (*n* = 42), primarily vegetated spaces and waterfront spaces. A smaller number examined university common spaces from an integrated perspective (*n* = 6), including vegetated spaces, waterfront spaces, plazas, courtyards, and sports fields, whereas another group examined a single campus space (*n* = 4), such as a courtyard, therapeutic garden, or pedestrian path. Sample sizes ranged from 12 to 1,236 because of differences in study settings and methods. Most studies surveyed university students without restricting the sample to a particular subgroup (*n* = 34), with a balanced gender distribution. Some studies distinguished academic levels and specifically surveyed undergraduates (*n* = 4), postgraduates (*n* = 1), or combined undergraduate and postgraduate samples (*n* = 6). Other studies restricted participants’ ages, including students aged over 18 years (*n* = 1), university students aged 18–23 years (*n* = 1), undergraduates aged 18–24 years (*n* = 1), and university students aged 18–26 years (*n* = 1). Several studies examined specific student groups, including full-time students (*n* = 1), students from different disciplines (*n* = 1), second-year landscape-architecture students (*n* = 1), and university students with educational backgrounds in architecture and urban–rural planning (*n* = 1). These participant groups partially overlapped (Table 2).

For the overall synthesis, a study was coded as “+” when an association between university common spaces and students’ psychological restoration was supported, “−” when the association was not supported, and “±” when the association was only partially supported or conclusions were contradictory across groups. Forty-six studies confirmed an association between university common spaces and university students’ psychological restoration; for example, common spaces with certain positive restorative characteristics significantly influenced psychological restoration (7, 17, 32). Six studies supported only selected associations between spatial characteristics and restoration. For example, the visibility and configuration of campus green spaces were not associated with students’ psychological restoration, whereas green-space area showed a significant positive association (33) (Table 2). Regarding effect pathways, 38 studies primarily examined the direct effects of university common spaces on students’ psychological restoration, whereas only 14 also showed that common spaces could affect restoration outcomes indirectly through mediators, principally physical activity, social interaction, and attitudes toward contact with landscape green spaces.

### 3.2 Content of the existing literature

In environmental psychology, spatial settings and their constituent elements are commonly conceptualized as environmental characteristics or exposures, whereas the psychological responses elicited by these environments are typically assessed as restoration outcomes. Accordingly, this review synthesized the existing literature from two complementary perspectives: (1) the restorative potential of different types of university common spaces and their constituent elements, as reflected in the environmental characteristics examined in relation to restoration; and (2) the psychologically restorative effects of university common spaces on university students, as reflected in the restoration outcomes assessed across the included studies.

#### 3.2.1 Environmental characteristics: restorative potential of university common space types and constituent elements

Although empirical studies of psychological restoration in university common spaces have increased in China and internationally in recent years, knowledge of the restorative effects of different common-space types remains limited. Most studies have focused only on the effects of campus landscape green spaces (*n* = 42), with research on university common spaces largely restricted to vegetated spaces and waterfront spaces. Some studies have focused on a specific campus space (*n* = 4), such as the restorative effects of a themed therapeutic garden. Only six studies emphasized the need to examine university common spaces from an integrated perspective, including the effects of vegetated spaces, waterfront spaces, plazas, courtyards, and sports fields considered together on students’ psychological restoration. Although most studies have focused on campus landscape green spaces, researchers have continued to ask which types of university common spaces provide the greatest restorative benefits, resulting in some comparative studies.

Many studies have confirmed that waterfront and vegetated campus green spaces can significantly promote positive emotions among university students (26, 34, 35). Contact with natural green spaces can effectively reduce students’ stress and anxiety and help maintain higher levels of positive emotions (22, 23, 36). Comparisons across common space types have shown significant spatial heterogeneity in restorative quality: waterfront spaces often provide greater restoration (15, 32, 37), followed by vegetated spaces, whose restorative effects are more pronounced than those of plazas and courtyards (38). Two recent studies also found considerable restorative potential in campus sports fields (17, 39). However, another empirical study found no clear difference in restoration across common-space types, with all types showing positive restorative characteristics: waterfront spaces, vegetated spaces, and commuting paths exhibited characteristics of being away, whereas sports fields exhibited compatibility (7).

Recent studies have paid greater attention to the restorative quality of constituent elements within university common spaces and have confirmed that these elements make different contributions to university students’ psychological restoration (18). Based on a synthesis of the relevant literature, the factors influencing restoration in university common spaces can be grouped into three categories: architectural-environmental, landscape-environmental, and facility-support elements. Architectural-environmental elements (*n* = 5) include the scale and openness of building enclosures and building-façade form; landscape elements (*n* = 51) include vegetation quantity, plant species, plant color, broad lawns, water features, and perceived naturalness; facility-support elements (*n* = 6) include the quantity, quality, and maintenance of facilities (Table 2).

##### 3.2.1.1 Restorative potential of architectural-environmental elements

Architectural-environmental elements are often overlooked in research on university common spaces; only five studies have examined their restorative quality. These elements can strengthen or weaken the restorative qualities of being away and extent within a common space, thereby promoting or impeding students’ psychologically restorative outcomes. For example, greater openness of building enclosures can elicit a sense of being away (18). This may be because enclosure is an important factor in students’ responses to an environment: greater spatial openness is more likely to evoke psychological experiences that differ from those associated with ordinary learning environments and to help students mentally distance themselves from exhausting settings (17). Historic building forms in common spaces can enhance environmental fascination and promote positive emotional connections between students and campus environments (17).

Two recent studies emphasized the relationship between building façades and psychological perception. Variation in façade form can elicit different emotional experiences; visually varied façades can attract attention and shift students’ attention away from routine learning contexts, thereby promoting restoration (17). In contrast, excessively dense façades may elicit negative emotions such as frustration, tension, and disappointment because high façade density reduces visual depth and reduces visual appeal, thereby reducing the restorative characteristic of being away (18). Other research found no significant effect of façade form on the regulation of university students’ negative emotions, whereas variation in building height played an important role. Building interfaces with varied heights increase visual richness, elicit a stronger sense of being away and greater fascination, and are positively associated with restoration (30, 40). A recent psychological experiment found that the presence of buildings negatively affected restorative potential and students’ positive approach behavior. However, changes in participants’ electroencephalographic activity further showed that the effects of buildings were not uniformly negative: varied building forms and more open enclosures had positive effects on students’ restoration (41). These findings provide indirect evidence that architectural-environmental elements influence university students’ psychological restoration and highlight the need for further research in this area.

##### 3.2.1.2 Restorative potential of landscape elements

Almost all studies (*n* = 51) examined the effects of landscape elements in university common spaces on university students’ psychological restoration. The following synthesis is organized around vegetation, water features, visual and spatial perceptual attributes, and soundscape, distinguishing physical characteristics from subjective environmental appraisals. Substantial evidence indicates that adequate vegetation, diverse plant species, and appropriate spatial distribution are significantly associated with students’ restoration (35, 37, 38). Specifically, sufficient vegetation cover can help university students alleviate the cognitive load associated with studying, while diverse vegetation can engage involuntary attention to a greater extent and reduce directed-attention fatigue (25, 42, 43). Plant species also influence restorative potential through variation in appearance: tree color, canopy size, and trunk form elicit different psychological responses, and trees with larger canopies can induce more positive emotions (11, 20, 44). Plant color significantly affects students’ positive emotions: green vegetation can promote relaxation (42, 43), yellow vegetation can increase inner calm (20), and red vegetation readily attracts visual attention (45). Broad lawns are also regarded as positive restorative elements (32, 46), but their restorative effect is weaker when they serve only an ornamental function because students cannot enter them for activities, reducing environmental compatibility (17). Other research has noted that large areas of lawn may satisfy students’ need for prospect but fail to satisfy their need for refuge (47).

Water features are often key constituent elements that increase the attractiveness and restorative potential of university common spaces. Several studies have shown that almost all students hold more positive attitudes toward common spaces containing water (12, 48, 49). Such spaces often receive higher restoration scores than spaces without water, and surveyed students typically report a stronger sense of psychological distance and greater improvement in positive emotions (26, 37, 42, 50). Water features with high aesthetic appeal are more attractive to university students seeking stress relief, and aesthetically pleasing waterscapes have been shown to be effective in reducing psychological stress and alleviating mental fatigue (17, 18, 32). Still water can provide a tranquil atmosphere that facilitates relaxation and calm (47), whereas moving water is a key element in improving the quality of university common spaces (36, 51). Combinations of water and vegetation can also improve comfort and strengthen restorative potential (15). Reflections in water may engage involuntary attention to a greater extent than the physical objects themselves and enrich the visual experience of the spatial environment (45).

Beyond vegetation and water features, studies have examined visual and spatial perceptual attributes, including perceived naturalness, green-space availability, visibility, accessibility, and attractiveness. Studies indicate that university students aged 17–27 years commonly regard nature as a means of escaping everyday life, and that perceived naturalness can help reduce anxiety, depression, and uncertainty (9, 16, 43). The natural characteristics perceived by students in common spaces, and the strength of that perception, can provide opportunities for restoration and enhance restorative experiences (7, 10, 14, 24, 35, 52). Green view ratio is discussed here because it quantifies greenery within the field of view rather than vegetation cover alone. It is treated as an objective indicator of visual exposure to greenery that may inform perceived naturalness, not as a direct measure of that subjective appraisal. The most favorable perceptions of restoration have been observed at a moderate green view ratio of 24–34%, whereas excessive greenery can obstruct views and elicit negative emotional responses (8, 23, 31, 53). Associations between perceived availability, visibility, accessibility, and attractiveness of campus landscape green spaces and students’ attention restoration and stress reduction have also been supported (32, 54). These subjective appraisals should be assessed alongside complementary physical indicators rather than replaced by them. Relevant physical characteristics include green-space quantity, vegetation coverage, and area (16, 33, 41, 55), as well as spatial configuration, location, and design features (22, 31, 44, 56, 57).

Soundscape represents a further dimension of the landscape experience. The sound of running water, for example, can stimulate thought and contribute positively to restoration (36, 51). A tranquil atmosphere in university common spaces has been positively associated with perceived restoration (13, 34, 58, 59). However, tranquility should not be equated with quietness alone: evidence from urban residential soundscapes suggests that it encompasses perceived restorativeness and pleasantness rather than merely low sound levels (60). A soundscape perspective may therefore provide a more comprehensive basis for interpreting the restorative potential of campus acoustic environments.

##### 3.2.1.3 Restorative potential of facility-support elements

Only six studies examined the effects of facility-support elements—amenities that support rest and activity—in university common spaces on university students’ psychological restoration. These elements mainly comprised rest facilities, such as seats, and activity facilities, such as sports fields and fitness equipment. Existing research suggests that students with greater restoration needs pay more attention to the quantity and quality of rest facilities in common spaces (11, 43). Sufficient, comfortable seating provides more opportunities for rest and better satisfies students’ desire for relaxation and stress relief (61). Rest facilities that provide readily accessible views can increase the time students spend resting in university common spaces, increase the duration and frequency of contact with nature, and create more opportunities for social interaction, thereby supporting emotion regulation and psychological restoration (45). Clean and well-maintained seats can also encourage students to rest to some extent (17).

Existing studies confirm that the maintenance, convenience, and diversity of activity facilities influence university students’ psychological restoration. In university common spaces, an adequate number of activity areas can encourage students to engage in physical exercise (44), while high-quality sports facilities can support feelings of pleasure and relaxation (11, 31). Ongoing maintenance of activity areas and fitness facilities can enhance environmental compatibility (44, 59), and convenient access to diverse activity areas can increase students’ enthusiasm for physical activity and significantly promote restoration (17). However, one study found that placing too many activity areas near teaching buildings may keep students mentally immersed in their routine learning environment during activities, making it difficult to elicit a sense of being away (18). Another study suggested that restoration in activity areas reflects the joint contribution of environmental characteristics and students’ activities (37). This distinction is particularly relevant to sports fields, where restoration may occur through passive or contemplative engagement with the setting, participation in physical activity, or their combination. Although ART is relevant to attentional recovery, it should not be restricted to passive exposure or assumed to explain all benefits of exercise. Hug et al. (62) found that perceived restorative quality predicted self-reported exercise frequency in both indoor and outdoor settings, highlighting the relationship between environmental appraisal and activity participation rather than establishing a causal effect of exercise on restoration (62). Evaluating sports fields therefore requires distinguishing environmental exposure from activity-related effects, considering activity type, intensity, duration, and social interaction rather than relying on visual assessments alone.

#### 3.2.2 Restoration outcomes: effects of university common spaces on university students’ psychological restoration

In studies examining the restorative effects of university common spaces, the conceptualization and interpretation of psychological restoration have been primarily informed by Attention Restoration Theory (ART) and Stress Reduction Theory (SRT). Within these theoretical frameworks, restorative effects may manifest both as improvements in broader psychological states, such as mental health and psychological well-being, and as specific process-level responses, including attention restoration, regulation of negative emotions, and stress reduction (4, 63). Based on the outcome levels captured by the measures and their conceptual proximity to the core construct of psychological restoration, this review classifies the measures into two categories: broader restoration-related outcome measures and psychological restoration process measures. The former capture overall mental health and psychological well-being associated with restorative environments, as well as broader outcomes reported in some studies, such as environmental satisfaction and quality of life. The latter assess specific psychological responses, including attention restoration, regulation of negative emotions, stress reduction, vitality restoration, and reduced loneliness. Importantly, environmental satisfaction and quality of life are treated as broader outcomes associated with perceived restorativeness rather than as direct measures of psychological restoration.

Of the 52 included studies, 13 examined broader restoration-related outcomes. Of these, 10 characterized the broader psychological benefits of restoration in terms of mental health (*n* = 5) and psychological well-being (*n* = 5), whereas three reported broader restoration-related outcomes, including environmental satisfaction (*n* = 1) and quality of life (*n* = 2). These two outcomes represent broader psychological outcomes that some studies reported alongside their assessments of restoration processes. The remaining 39 studies primarily evaluated psychological restoration using specific process measures. Some studies assessed restorative outcomes using multiple measures in combination. The most frequently used measures were regulation of negative emotions (*n* = 20), attention restoration (*n* = 14), and stress reduction (*n* = 12), followed by vitality restoration (*n* = 2) and reduced loneliness (*n* = 1) (Figure 5; Table 3). Among the studies using restoration process measures, most examined only a single dimension of restoration (*n* = 29), such as regulation of negative emotions or attention restoration, whereas a smaller number integrated multiple process measures to evaluate restorative effects (*n* = 10).

**Figure 5:**
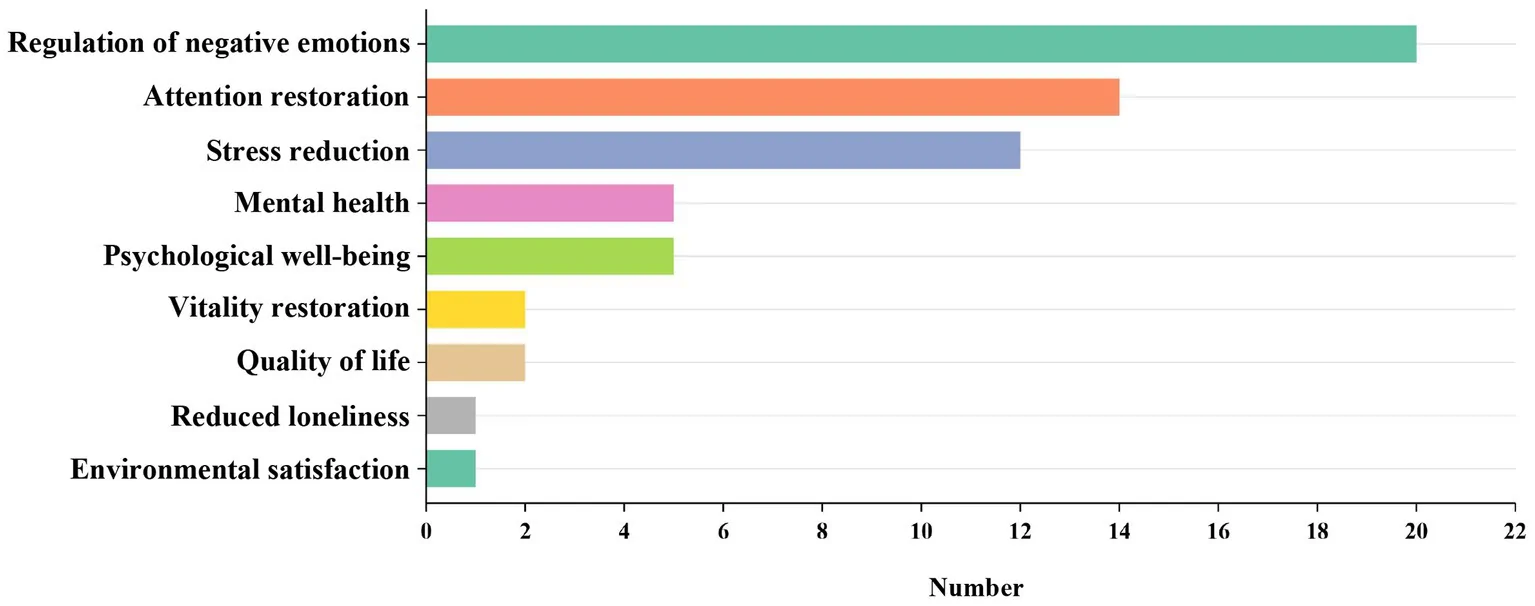
Distribution of indicators used to measure psychological restoration.

**Table 3:** Principal tools used to measure students’ psychologically restorative outcomes.

Categories and counts	Psychological restoration	Measurement types	Measurement instruments	Representative content	References
mental health (*n* = 5); psychological well-being (*n* = 5); environmental satisfaction (*n* = 1); quality of life (*n* = 2)	mental health	validated scale (used directly or adapted)	Warwick–Edinburgh Mental Well-being Scale (WEMWBS)	Hope; activity; belonging; relationships.	(12)
	mental health	validated scale (used directly or adapted)	WHO-5 Well-Being Index (WHO-5)	Feeling cheerful; feeling relaxed; feeling active; feeling well-rested; and feeling that life is meaningful and interesting.	(33)
	mental health	validated scale (used directly or adapted)	WHO-5 Well-Being Index (WHO-5)	Feeling calm and relaxed; feeling happy and satisfied; feeling well-rested; feeling energetic; and feeling vital.	(66)
	mental health	validated scale (used directly or adapted)	WHO-5 Well-Being Index (WHO-5)	Feeling calm and relaxed; feeling happy and satisfied; feeling well-rested; feeling energetic; and feeling vital.	(35)
	mental health	validated scale (used directly or adapted)	WHO-5 Well-Being Index (WHO-5)	Feeling cheerful; feeling relaxed; feeling active; feeling well-rested; and feeling that life is meaningful and interesting.	(44)
	psychological well-being	validated scale (used directly or adapted)	WHO-5 Well-Being Index (WHO-5)	Feeling cheerful; feeling relaxed; feeling active; feeling well-rested; and feeling that life is meaningful and interesting.	(22)
	psychological well-being	self-developed questionnaire	five-point Likert scale	Age, educational level, gender, and questions about resting practices.	(23)
	psychological well-being	validated scale (used directly or adapted)	Perceived Stress Scale (PSS)	Students’ well-being, ranging from low (very unhappy / somewhat unhappy / neither happy nor unhappy) to high (very happy / somewhat happy).	(61)
	psychological well-being	self-developed questionnaire	five-point Likert scale	Resting in Hof Garden gives me the energy needed to relieve academic stress; after resting in Hof Garden, I feel more satisfied and balanced; after resting in Hof Garden, I can concentrate better.	(57)
	psychological well-being	self-developed questionnaire	five-point Likert scale	What is your favorite activity in campus green spaces? What are your purposes and feelings when visiting green spaces? How frequently do you visit them?	(50)
	environmental satisfaction	validated scale (used directly or adapted)	Semantic Differential Scale (SDS)	Not oppressive at all–extremely oppressive; very enclosed–very open; very dissatisfied–very satisfied.	(30)
	quality of life	validated scale (used directly or adapted)	WHOQOL-BREF; Perceived Restorativeness Scale (PRS)	Twenty-six items cover the physical (e.g., How is your mobility?), environmental (e.g., How much opportunity do you have for leisure activities? How healthy is your physical environment?), psychological (e.g., How well can you concentrate?), and social (e.g., How satisfied are you with the support provided by your friends? Does resting here help you detach from everyday work) domains of quality of life.	(24)
	quality of life	validated scale (used directly or adapted)	WHOQOL-BREF; Perceived Restorativeness Scale (PRS)	Here I can relax my attention; here I feel a sense of belonging; here I can think and organize things easily.	(55)
attention restoration (*n* = 14)	attention restoration; stress reduction	validated scale (used directly or adapted)	Perceived Restorativeness Scale (PRS)	This environment helps me get away from the pressures and worries of studying; there are many different areas here; the environment is fascinating and captures my attention.	(18)
	attention restoration	self-developed questionnaire	five-point Likert scale	How satisfied are you with the selected space as an attention-restoration space? What are your visit frequency, visit time, length of stay, activities, and whether you are accompanied? If you want to find a place to relax your mind, which campus environments do you consider suitable?	(32)
	attention restoration; stress reduction	self-developed questionnaire	seven-point Likert scale	Based on your actual visiting experience, to what extent does this place meet your needs for stress relief and mental restoration?	(7)
	attention restoration	validated scale (used directly or adapted)	Restoration Outcome Scale (ROS)	Three items address feelings of relaxation, calm, and enthusiasm; one assesses attention restoration, and two focus on clearing away worries and thoughts.	(16)
	attention restoration	validated scale (used directly or adapted)	Perceived Restorativeness Scale (PRS)	Here my thoughts are free from stress; I can take time away from my busy life; I can consider everything at once or nothing at all; I can put all my problems aside; I feel closely connected with nature.	(40)
	attention restoration; stress reduction	self-developed questionnaire	five-point Likert scale	Each measured variable contains 3–4 items, with seven measured variables in total.	(17)
	attention restoration	validated scale (used directly or adapted)	Restoration Outcome Scale (ROS)	I feel mentally clear; I feel enthusiastic and energetic about everyday life; I feel focused and alert; I feel calm and clear-headed; I can forget everyday worries.	(45)
	attention restoration; stress reduction; regulation of negative emotions	validated scale (used directly or adapted)	Perceived Restorativeness Scale (PRS)	The scale includes 22 items: 5 items for being away, 5 for fascination, 6 for compatibility, and 5 for extent.	(37)
	attention restoration	validated scale (used directly or adapted)	Perceived Restorativeness Scale (PRS)	Being away: I like going to places like this to stop thinking about things I have to do; coherence: it is easy to see how this place is organized; scope: this place is large enough to explore in many directions; fascination: my attention is drawn to many interesting things here.	(39)
	attention restoration	validated scale (used directly or adapted)	Perceived Restorativeness Scale (PRS); Restorative Components Scale (RCS)	The 26-item scale measures being away, fascination, extent, and compatibility to determine students’ perceived restoration in different green spaces.	(38)
	attention restoration; regulation of negative emotions	validated scale (used directly or adapted)	Self-Assessment of Restorative Impact scale (SARI)	Eight items cover emotional, physiological, cognitive, and behavioral responses (e.g., How would you describe your level of anxiety in the CGS?).	(35)
	attention restoration	qualitative description	interview guide	Which environmental elements on campus make you feel relaxed and relieve stress? Which make you feel tense, anxious, irritated, or stressed?	(46)
	attention restoration; regulation of negative emotions	self-developed questionnaire	five-point Likert scale	To what extent did you hear the following sounds in the current environment?	(51)
regulation of negative emotions (*n* = 20)	regulation of negative emotions	self-developed questionnaire	two-point scale	Does this scene make you feel depressed, disappointed, happy, focused, or relaxed?	(31)
	regulation of negative emotions	qualitative description	interview guide	Time spent in the therapeutic garden; describe your experience and the sensory activities you used (e.g., sitting in reflection, meditating, experiencing nature, reading, studying, interacting with others, and planting or harvesting herbs in the garden).	(11)
	regulation of negative emotions	validated scale (used directly or adapted)	Profile of Mood States (POMS); Perceived Restorativeness Scale (PRS)	Greater variation between enclosed and open spaces, with greater emotional fluctuation; smaller variation, with more stable emotions.	(47)
	regulation of negative emotions	validated scale (used directly or adapted)	Positive Affect scale (PANAS-P)	I feel energetic; I feel enthusiastic; I feel cheerful; I feel happy; I feel excited; I feel proud; I feel inspired; I feel strong; I feel grateful.	(35)
	regulation of negative emotions; stress reduction	validated scale (used directly or adapted)	Restorative Components Scale (RCS)	Being away: I do things here that are different from what I usually do (4 items); Extent: The elements here are interconnected (4 items); Fascination: There are many things here that attract my attention (4 items); Compatibility: The environment here provides opportunities for me to engage in activities I enjoy (4 items).	(15)
	regulation of negative emotions	self-developed questionnaire	five-point Likert scale	Life satisfaction; tension and anxiety; loneliness; social connection; ability to concentrate; and current stress level.	(25)
	regulation of negative emotions	validated scale (used directly or adapted)	Place Attachment Inventory (PAI); Positive and Negative Affect Schedule (PANAS); Landscape-Specific Affect Bonding Scale (LSABS)	When I am here, I feel relaxed; I feel happiest here; this is my favorite place; when I have been away too long, I really miss this place; I feel that I can truly be myself here; this is the best place to do what I enjoy most (21 items in total).	(45)
	regulation of negative emotions	qualitative description	interview guide	How did you feel during the walk? Did anything noteworthy happen during the walk?	(10)
	regulation of negative emotions	qualitative description	interview guide	If you had one hour of free time, where on campus would you most like to go? What emotional experiences would you have after visiting the place and engaging in the relevant activities?	(58)
	regulation of negative emotions	validated scale (used directly or adapted)	Positive and Negative Affect Schedule (PANAS); Perceived Restorativeness Scale (PRS)	Feelings of interest, excitement, intensity, enthusiasm, and pride.	(41)
	regulation of negative emotions	qualitative description	interview guide	How do you relieve stress outdoors on campus? Do you associate outdoor campus activities with stress release? Have you had any particularly negative or positive experiences during outdoor campus activities? Where on campus do you feel best? Please describe your ideal university campus outdoor environment.	(13)
	regulation of negative emotions	validated scale (used directly or adapted)	Perceived Restorativeness Scale (PRS); Profile of Mood States (POMS)	I feel connected to the natural environment; I can make space to think about problems.	(6)
	regulation of negative emotions	validated scale (used directly or adapted)	Profile of Mood States (POMS)	The questionnaire uses 37 adjectives to assess six emotional dimensions: tension, depression, anger, vitality, fatigue, and confusion, including restlessness, irritation, exhaustion, alertness, and inability to concentrate.	(9)
	regulation of negative emotions	qualitative description	interview guide	Was the effect of the photographed place on emotion positive or negative? Which campus location makes you happier? Do you prefer places with open views?	(14)
	regulation of negative emotions	validated scale (used directly or adapted)	Perceived Restorativeness Scale (PRS); Positive and Negative Affect Schedule (PANAS)	Perceived Restoration Emotional: I feel relaxed when viewing the scene presented. Physiological: My breathing becomes rapid. Cognitive: I feel focused on the scene presented. Behavioral: I want to stay here longer. Positive and Negative Affect Positive affect: I feel determined when viewing the scene presented (4 items). Negative affect: I feel bored when viewing the scene presented (4 items).	(36)
	regulation of negative emotions	self-developed questionnaire	five-point Likert scale	Positive affect; negative affect; health satisfaction; and interpersonal-relationship satisfaction.	(54)
	regulation of negative emotions	validated scale (used directly or adapted)	short-form Positive and Negative Affect Schedule (PANAS-SF); short-form Perceived Restorativeness Scale (PRS-SR)	Five positive-affect indicators and five negative-affect indicators; four dimensions—emotional, physiological, cognitive, and behavioral—with eight evaluation indicators.	(49)
stress reduction (*n* = 12)	stress reduction	qualitative description	interview guide	Descriptions of participants’ psychological or physiological states, including relaxation and calm, escape and perspective shifting, presence and mindfulness, positive emotions, and negative emotions.	(34)
	stress reduction	qualitative description	interview guide	Well-being; stress reduction; enhanced self-esteem; and overall life satisfaction.	(19)
	stress reduction	validated scale (used directly or adapted)	short-form revised Restoration Scale (ARAS-R)	Emotional: I feel relaxed in response to the presented scene; physiological: my breathing becomes rapid; cognitive: I feel focused on the presented scene; behavioral: I want to stay here longer.	(26)
	stress reduction; regulation of negative emotions	validated scale (used directly or adapted)	Depression Anxiety Stress Scales-42(DASS-42)	Difficulty relaxing; nervousness; being easily upset or agitated; irritability and overreaction; and impatience.	(48)
	stress reduction	self-developed questionnaire	five-point Likert scale	Relaxing and relieving fatigue; concentrating on a specific thought; and calming negative emotions.	(42)
	stress reduction	validated scale (used directly or adapted)	State–Trait Anxiety Inventory (STAI); Perceived Restorativeness Scale (PRS)	STAI-S comprises 20 items (e.g., tense and relaxed); PRS comprises 20 items (e.g., high being away, fascination, extent, and compatibility).	(20)
	stress reduction; regulation of negative emotions	validated scale (used directly or adapted)	Profile of Mood States (POMS)	Positive- and negative-affect indicators.	(53)
vitality restoration (*n* = 2) reduced loneliness (*n* = 1)	vitality restoration	self-developed questionnaire	four-point scale	After examinations and intensive study, when you feel mentally exhausted and unable to concentrate, does this environment help restore your vitality? If you stay here for 20 min, do you feel (1) well-rested and (2) that your vitality has returned?	(43)
	attention restoration; reduced loneliness; vitality restoration	validated scale (used directly or adapted)	Restoration Outcome Scale (ROS); Restorative Components Scale (RCS)	Direct attention restoration (1 item); clearing mental clutter (2 items); relaxation and calm (3 items); novelty (3 items); escape (4 items); fascination (6 items); extent (4 items); and compatibility (5 items).	(8)

##### 3.2.2.1 Measures of broader restoration-related outcomes

In studies that characterized broader restorative benefits in terms of mental health and psychological well-being, commonly used items included feeling “calm or relaxed,” “happy or content,” and “energetic and vigorous.” These items were primarily used to assess whether university students experienced positive affect, stress relief, and physical relaxation (22, 33, 44, 45, 56). Several studies further suggested that restorative environments intended to promote students’ psychological well-being should be located close to their primary sources of stress, such as their places of study, thereby providing readily accessible opportunities for psychological restoration (23, 57, 61). In addition, two studies examined the relationship between perceived restorativeness and quality of life (24, 55). Both reported significant indirect effects of environmental characteristics on quality of life through perceived restorativeness, suggesting that restorative experiences may represent a potential psychological pathway through which environmental characteristics are related to university students’ quality of life. Another study found that perceived restorativeness was associated with satisfaction with the spatial environment. Students’ environmental satisfaction increased as their psychological needs were met, and those reporting greater environmental satisfaction also tended to report higher quality of life and better psychologically restorative outcomes (30). Overall, common spaces rated more favorably by students appeared to provide greater perceived opportunities for restoration and were also associated with better quality of life.

##### 3.2.2.2 Measures of psychological restoration processes

Across 39 studies, restoration outcomes were assessed using indicators derived from Attention Restoration Theory and Stress Reduction Theory, including attention restoration, stress reduction, regulation of negative emotions, vitality restoration, and reduced loneliness. Data were collected using established scales, adapted validated scales, self-developed questionnaires, or interview guides.

###### 3.2.2.2.1 Attention restoration

Attention restoration is an important criterion for evaluating the effectiveness of restorative environments (3). Kaplan proposed Attention Restoration Theory (ART) in 1983 as a cognitive framework for restorative environments. The theory identifies four restorative components—being away, fascination, extent, and compatibility—which provide an important basis for assessing the restorative potential of spatial environments (63). In learning environments, sustained concentration and prolonged use of directed attention reduce university students’ ability to inhibit distraction and maintain focus (7). Restorative environments can help students restore depleted attentional resources and elicit positive psychological responses (32, 37). Multiple studies have emphasized the key role of constituent elements of university common spaces in attention restoration (18); the layout and design of these elements significantly affect students’ ability to concentrate (16). For example, building-height differences, tree height, and shrub area are significantly associated with attention restoration and psychological restoration (39, 40).

###### 3.2.2.2.2 Stress reduction

Stress Reduction Theory (SRT), proposed by Ulrich in the field of healthcare architecture, holds that a manifest state of stress can impair cognitive and behavioral functioning, whereas environments containing positive features can make individuals feel relaxed and less stressed (64). University common spaces can help students reduce psychological stress by providing opportunities for rest and vitality restoration (48), and by facilitating the use of common-space facilities and contact with landscape environments and nature (19). Research has also shown that more trees, lawns, and diverse vegetation in common spaces can engage involuntary attention to a greater extent while reducing students’ psychological stress (20). Students who frequently participate in common-space activities in positive ways report higher levels of restoration and lower perceived stress (50).

###### 3.2.2.2.3 Regulation of negative emotions

Multiple studies have emphasized that university common spaces influence university students’ subjective emotional experiences. When environmental elements meet students’ psychological expectations or needs, they elicit positive emotional responses (10, 11, 25). For example, well-planned campus green spaces can lower students’ blood pressure and significantly increase positive emotions (15), while sky visibility and building façades can induce different emotional experiences (31). Contact with waterside landscapes helps students rapidly alleviate negative emotions and promotes restoration (9). Accordingly, creating spaces that alleviate negative emotions is an important approach to designing restorative campus environments (13, 14, 58).

###### 3.2.2.2.4 Vitality restoration and reduced loneliness

Vitality restoration and reduced loneliness have also been used as indicators of university students’ psychological restoration and psychological well-being (22, 23). The two outcomes are closely and positively related. Restored vitality can improve negative emotional states, make students more willing to initiate contact with others, and thereby reduce loneliness. Conversely, reduced loneliness can alleviate psychological anxiety and depression and thus promote vitality restoration (55). Existing research suggests that the design of university common spaces should effectively support vitality restoration and reduce loneliness, providing university students with more opportunities for psychological restoration (57).

### 3.3 Pathways linking university common spaces and students’ psychological restoration

Based on the existing literature, this study also used the independent variable–mediator–dependent variable framework to elucidate the potential pathways underlying the psychologically restorative effects of university common spaces (65). This framework describes the sequence of “spatial characteristics–mediating factors–psychological restoration.” Specifically, the environmental characteristics of university common spaces, represented by the independent variable X, may be associated with psychologically restorative outcomes, represented by the dependent variable Y, through their influence on the mediator M. Here, M may serve as a potential mediator through which university common spaces exert psychologically restorative effects, and the process by which X influences Y via M constitutes the mediating pathway (Figure 6).

**Figure 6:**
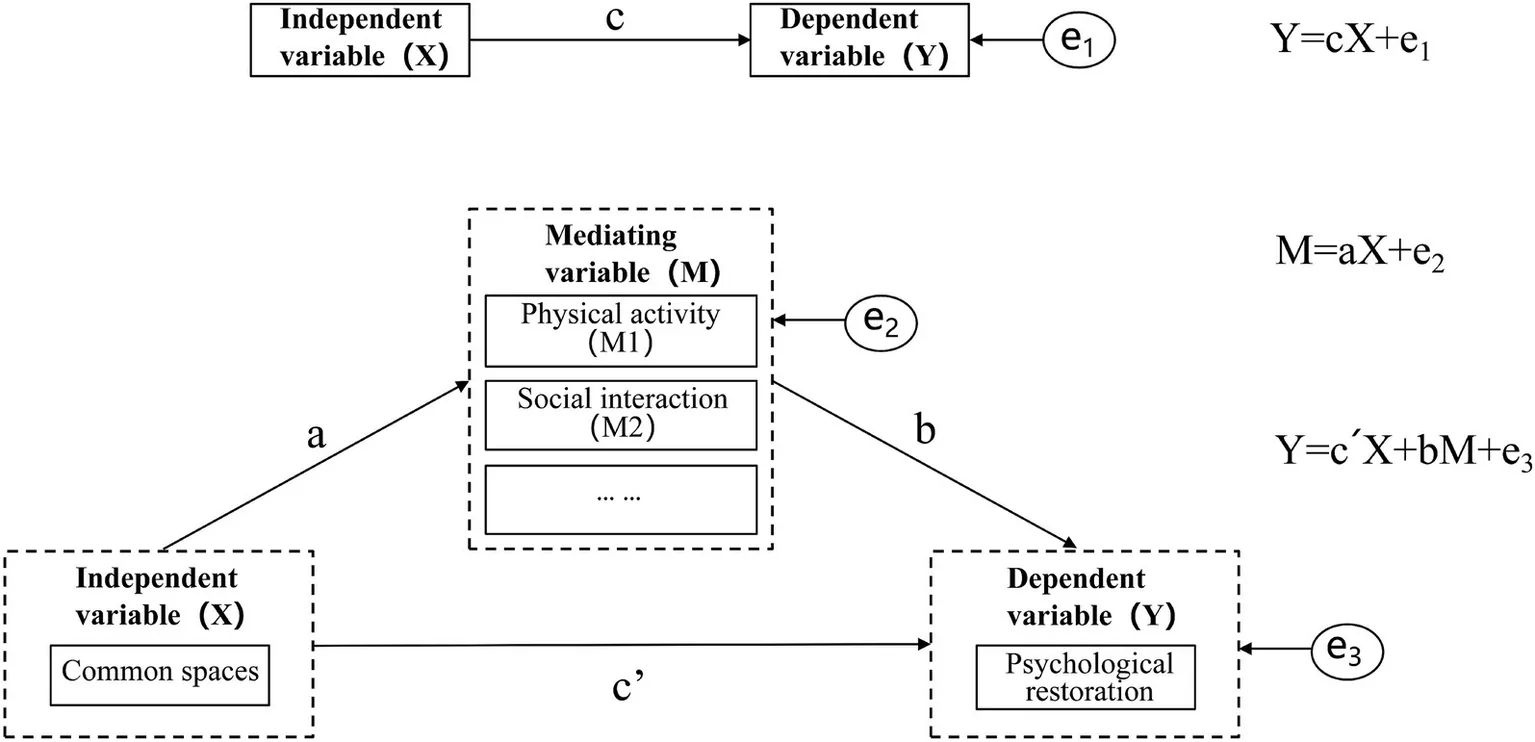
Schematic illustration of the mediation model (65).

Based on this framework, the potential pathways through which the environments of university common spaces influence university students’ psychological restoration can be classified into two types: (1) a direct pathway, “characteristics of university common spaces → university students’ psychological restoration”; (2) an indirect pathway, “characteristics of university common spaces → mediating factors → university students’ psychological restoration”. Existing studies have focused mainly on direct associations, and only a small number have further examined indirect pathways.

#### 3.3.1 Direct pathway linking university common spaces and students’ psychological restoration

A direct pathway refers to the process by which the characteristics and constituent elements of university common spaces directly influence university students’ psychological restoration. Research in environmental psychology indicates that perceptual experiences can promote restoration by directly influencing attention and emotion. In particular, visual experiences of spatial environments can engage involuntary attention, redirect directed attention, and directly promote restoration (63). Characteristics of university common spaces, such as visual complexity and sky visibility; spatial elements, such as buildings, plants, and water; and attributes of spatial elements, such as quantity, scale, layout, and type, constitute the physical basis of spatial perception and are directly associated with university students’ restoration (11, 19, 31, 42, 48). For example, visual complexity and sky visibility in university common spaces can directly affect students’ restorative experiences, with higher visual complexity and sky visibility associated with greater restoration (18, 30, 41). Viewing historic buildings in university common spaces can elicit pleasant visual experiences and directly promote positive emotions (17). Green spaces near teaching areas, diverse tree species, and tree–shrub combinations can directly promote students’ restoration and evoke positive emotions such as happiness and vitality (38, 57).

#### 3.3.2 Indirect pathway linking university common spaces and students’ psychological restoration

An indirect pathway refers to the process by which characteristics and constituent elements of university common spaces influence university students’ psychological restoration through mediating factors. Current mediating pathways mainly involve attitudes and behavioral support.

##### 3.3.2.1 Indirect pathway based on attitudes

The attitude-based mediating pathway refers to the process by which university students’ subjective evaluations or emotional responses to the environment indirectly influence restoration. Existing studies have examined subjective evaluations of restorativeness and satisfaction, personal preferences, and attachment after contact with campus green spaces (Table 2). The sensory characteristics of university common spaces give rise to direct psychological experiences that shape students’ evaluations of perceived restorativeness and, in turn, indirectly contribute to restorative experiences (24). For example, perceived greenness in university common spaces has a significant positive effect on students’ psychological restoration and quality of life through the mediating effects of perceived restorativeness and satisfaction (8, 55). Objective greenness in the environment increases perceived greenness, provides students with opportunities for restoration, and elicits positive evaluations of restorativeness and satisfaction, thereby indirectly promoting restoration and improving quality of life (22, 36). Place attachment and landscape preference can also significantly mediate the effect of university common spaces on students’ restoration; however, the joint mediating effect of place attachment and landscape preference has not been replicated at a different study site (35).

##### 3.3.2.2 Indirect pathway based on behavioral support

The behavioral-support mediating pathway refers to the process by which common-space environments indirectly influence restoration by providing greater support for students’ activities. Although relatively few studies have examined this pathway, it has received increasing attention. The principal behavioral mediators are physical activity and social interaction.

###### 3.3.2.2.1 Physical activity

Research indicates that restoration often occurs during physical activity various forms and intensities. For university students who engage in prolonged cognitive work and sedentary behavior, physical activity is an important means of relieving stress (7). Characteristics of university common spaces have a significant positive effect on psychological restoration through the mediating role of students’ physical activity (33). For example, the openness, appropriate scale, and form of building enclosures can encourage students to participate in physical activities such as ball games and running. Attractive water features can encourage physical exercise and enhance psychological restoration through this behavioral mediation (17). Other research has shown that sky openness in university common spaces significantly affects both students’ sports participation and restoration, and that its effect on psychological restoration is fully mediated by exercise (66). This may be because sky openness is generally related to the number and size of sports fields, plazas, and courtyards. Common spaces with greater sky openness typically contain more open areas, lower building density, and greater spacing between structures, thereby increasing opportunities for physical activity and indirectly promoting psychological restoration (66).

###### 3.3.2.2.2 Social interaction

University common spaces provide places and opportunities for interaction among students, helping to increase the frequency of social activities, strengthen cohesion, and further promote restoration. However, only two empirical studies have demonstrated a significant mediating effect of social interaction between the characteristics of university common spaces and students’ psychological restoration. Attractive water features and higher vegetation coverage can alleviate the heat-island effect, promote social activity, and further affect restoration outcomes through social interaction (21). Sufficient rest facilities and facilities that provide views can increase students’ opportunities for social interaction and thereby promote restoration (21). Other research has used cohesion as a mediator and shown that contact with campus green spaces can promote restoration by strengthening social cohesion. For example, subjective measures of green-space exposure in university common spaces can directly affect social cohesion and subsequently influence students’ restoration through social cohesion (33).

### 3.4 Methods used in existing studies

Among the 52 included studies, approaches to measuring students’ restoration fell into three categories: (1) adapted validated scales or selected items from validated scales (*n* = 32); (2) self-developed measurement scales (*n* = 12); (3) descriptive material obtained through interviews in qualitative and mixed-methods studies (*n* = 8) (Figure 7; Table 3).

**Figure 7:**
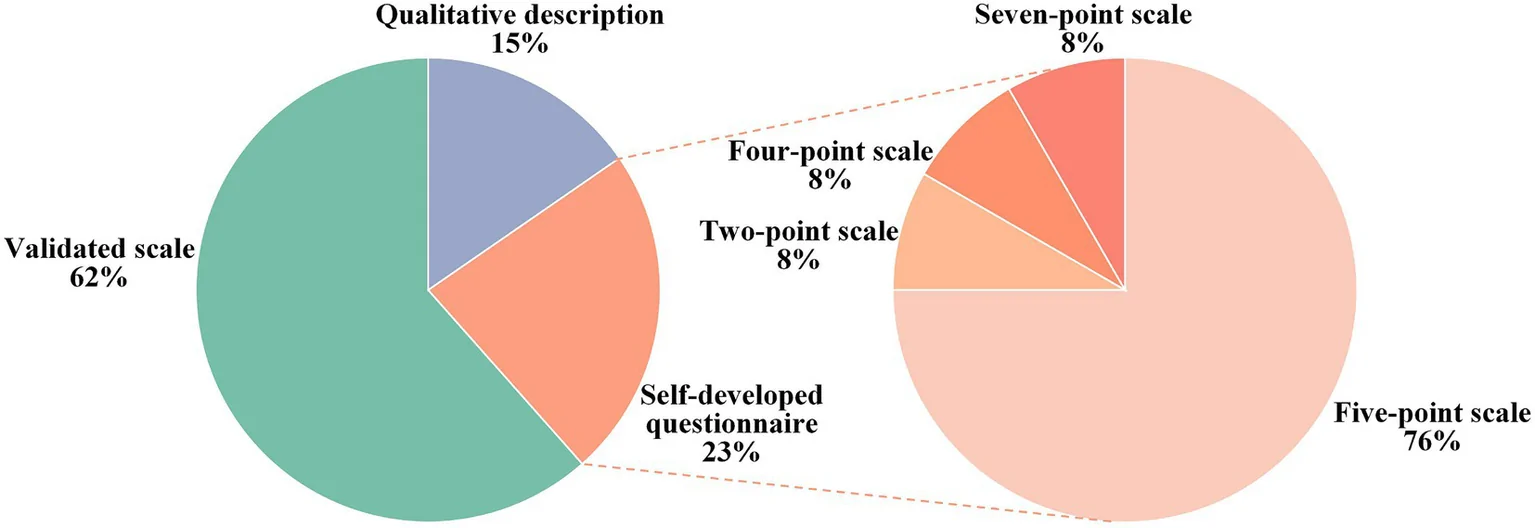
Distribution of instruments used to measure psychological restoration.

Psychological restoration was generally measured directly using validated scales, either in their original or adapted form; a small number of studies used self-developed questionnaires. For example, the WHO-5 Well-Being Index, Warwick–Edinburgh Mental Well-being Scale (WEMWBS), and Perceived Stress Scale (PSS) were used to assess restoration-related mental health and psychological well-being (12, 33, 44), the WHOQOL-BREF and Perceived Restorativeness Scale (PRS) were used to assess quality of life (24, 55), and the Semantic Differential Scale (SDS) was used to measure environmental satisfaction in university common spaces (30).

Studies assessing regulation of negative emotions mainly combined experiments with questionnaire surveys or qualitative interview guides. Common questionnaires included validated scales used directly or in adapted form, such as the Positive and Negative Affect Schedule (PANAS), Profile of Mood States (POMS), and Perceived Restorativeness Scale (PRS) (35, 45). Subjective questionnaire ratings were generally used to assess emotional valence, or preference for an emotional state (41, 45), whereas physiological indicators such as heart rate and electrodermal activity were generally used to assess emotional arousal (6, 9).

Attention restoration was primarily measured using questionnaire surveys and validated scales used directly or in adapted form, including the Perceived Restorativeness Scale (PRS), Restoration Outcome Scale (ROS), and Restorative Components Scale (RCS). A small number of studies also used self-developed questionnaires alongside established instruments (7, 32, 67). The PRS and RCS assess being away, fascination, extent, and compatibility to identify the characteristics of university common spaces that promote attention restoration and stress reduction, thereby measuring the influence of spatial environments on psychological restoration (68). The ROS primarily assesses restoration through direct attention restoration, clearing mental clutter, and relaxation and calm (8, 59).

Stress reduction was mainly measured using established, validated scales, including the Depression Anxiety Stress Scales-42 (DASS-42) (48), State–Trait Anxiety Inventory (STAI) (20), Perceived Restorativeness Scale (PRS), and revised short-form Restoration Scale (ARAS-R) (26). Some studies also used qualitative interview guides (19, 34) and self-developed questionnaires (42, 50).

Vitality restoration and reduced loneliness were mainly measured using adapted validated scales and self-developed questionnaires. For example, one study adapted the Restoration Outcome Scale (ROS) by adding measures of vitality and loneliness to assess whether students’ vitality was restored, loneliness was reduced, and interaction with classmates increased (8). Another study used a self-developed item—“If you stay in this environment for 20 min, do you feel that your vitality has been restored?”—to assess vitality restoration in a specific campus space (43).

## 4 Discussion

This scoping review synthesized evidence from 52 included studies on the relationship between university common spaces and university students’ psychological restoration. Overall, the existing research consistently supports the restorative potential of university common spaces, but the current evidence base has four main limitations: (1) research on environmental characteristics has been dominated by studies of campus green spaces and localized landscape elements, with insufficient attention to the integrated assessment of common-space types and the systematic assessment of architectural, landscape, and facility elements; (2) research on restoration outcomes has often relied on a single restoration indicator, without fully capturing the multidimensional nature of psychological restoration; (3) pathways of influence have mostly been interpreted as direct associations between environmental characteristics and restoration outcomes, while indirect mechanisms remain underexplored; (4) methods for identifying and quantifying spatial elements lack operational feasibility, limiting the translation of findings into campus design practice.

### 4.1 Insufficient research on the psychologically restorative potential of different types of university common spaces and their constituent elements considered systematically

Most existing studies have focused on the psychologically restorative potential of individual campus spaces and specific elements, with insufficient examination of how common-space characteristics and constituent elements collectively influence students’ restoration. For example, research has concentrated on vegetated and waterfront spaces, whereas evidence separately evaluating plazas, courtyards, and sports fields remains limited within the included studies (12, 19, 48). However, this apparent imbalance may partly reflect classification practices, as studies labeled “campus green space” may encompass settings within or adjacent to plazas, courtyards, or quadrangles without distinguishing their specific contributions. The gap therefore concerns limited spatially differentiated evidence rather than a demonstrated absence of research on these settings. Research on public urban squares also indicates that restorative opportunities are not confined to predominantly green environments (69). Future campus studies should report spatial configurations and use patterns explicitly and distinguish the contributions of vegetation, built features, and activities within mixed settings. Some studies have continued to adopt a traditional binary framework contrasting natural and built environments, focusing on restoration assessments and outcome comparisons across common-space types. This approach may lead to the simplistic conclusion that natural landscape types, such as vegetated and waterfront spaces, promote restoration, whereas built types, such as plazas, courtyards, and sports fields, impede it. It overlooks the possibility that well-designed built environments can also promote positive restorative experiences through open views, appropriate scale, varied interfaces, convenient facilities, and activity support (11, 30, 66).

Perceived safety and visibility may help explain why greenery does not consistently support restoration: dense vegetation can obstruct sightlines and heighten perceived threat, particularly after dark. In a Swedish study, Zeng et al. (70) found that trees were positively associated with self-identified tranquil scenes during daylight but negatively associated with such scenes at night (70). Although this evidence is not campus-specific and does not establish safety as the causal mechanism, it suggests that the benefits of greenery may depend on lighting conditions and safety perceptions. Perceived safety may therefore condition the attitude-based pathway described in Section 3.3.2, potentially shaping how visibility and vegetation relate to preference, place attachment, and restorative experiences. Future campus studies should test these relationships across daytime and nighttime conditions, considering vegetation density, sightlines, and lighting together.

In addition, existing studies of common-space elements have focused mainly on the effects of individual landscape elements, while evidence for architectural-environmental and facility-support elements is much less abundant (18, 21). Current research therefore cannot adequately evaluate the restorative potential of university common spaces as a whole. Future studies should move beyond single natural elements and examine the interactions among architectural, landscape, and facility-support elements within a systematic framework for university common spaces.

### 4.2 Limited application of existing integrative frameworks and multidimensional measurement in university campus contexts

This review classified the relevant measures into two categories: broader restoration-related outcome measures and psychological restoration process measures. The former included mental health, psychological well-being, and, in some studies, environmental satisfaction and quality of life; the latter included attention restoration, stress reduction, regulation of negative emotions, vitality restoration, and reduced loneliness. Importantly, environmental satisfaction and quality of life were considered broader outcomes associated with perceived restorativeness rather than direct measures of psychological restoration. Although the two categories represent different levels of outcome, their specific measurement content partly overlaps. For example, mental health and psychological well-being were often assessed using items related to affective states, stress, and vitality (22, 30, 44, 55). This overlap indicates a lack of consensus regarding the operational boundaries of psychological restoration and the relationships among outcomes at different levels in research on university common spaces.

This limitation does not imply that integrative theoretical frameworks are lacking in restorative-environment research involving general populations. Previous studies have integrated environmental characteristics, restorative experiences, and health outcomes at multiple levels. Hartig et al. (71) conceptualized the relationship between natural environments and health in terms of interrelated pathways involving stress reduction, physical activity, social connections, and environmental exposures, emphasizing that multiple mechanisms may operate simultaneously (71). Weber and Trojan (72) further demonstrated that both natural elements and built environments may have restorative potential and that environmental characteristics, activity patterns, social factors, and individual differences may jointly shape restorative experiences (72). Bornioli and Subiza-Pérez (73) proposed a multilevel model for urban built environments that distinguishes among containment, passive restoration, and active restoration and links physical and symbolic environmental characteristics to stress reduction, affective improvement, attention restoration, and broader well-being outcomes (73). Thus, the principal limitation of current research is not the absence of integrative frameworks for general populations, but their limited application, adaptation, and empirical testing in university campus contexts.

The university campus context may influence how these broadly applicable restorative pathways manifest and their relative strengths. First, university students are routinely exposed to sustained demands on directed attention, coursework, academic evaluation, and study-related stress; consequently, their restorative needs are closely linked to academic activities. Second, students’ exposure to university common spaces is typically frequent, brief, and embedded in their daily routines, often occurring between classes, during study breaks, or while moving around campus rather than during deliberately scheduled leisure activities. Third, university common spaces commonly serve multiple functions, including circulation, study, solitude, and social interaction. Their restorative effects may therefore be jointly influenced by noise, crowding, accessibility, support for activities, and the social context. These characteristics do not necessarily indicate that university students have fundamentally different restorative mechanisms from the general population, but they may alter the conditions under which these mechanisms are activated, their relative strengths and time scales, and their interactions.

Among the 39 studies that used psychological restoration process measures, most relied on a single restorative indicator to assess psychological restoration in university students. For example, some studies focused exclusively on the effects of university common spaces on students’ affective states and consequently inferred psychological restoration from reductions in negative affect (14, 25). Such an approach overlooks the multidimensional nature of psychological restoration and the potential interactions among attentional, stress-related, and affective processes (21). Future research should therefore build on existing integrative models, test their applicability and boundary conditions in university campus contexts, and combine multidimensional measures of attention, stress, affect, and vitality to clarify the interplay among restorative processes and how these processes manifest in university common spaces.

### 4.3 Limited empirical testing of restorative pathways in university campus contexts

Existing studies have generally reported significant associations between the types and attributes of constituent elements of university common spaces and psychological restoration among students. However, empirical explanations of how these spaces produce restorative effects remain limited. For example, the association between university common spaces and lower psychological stress may reflect a direct environmental effect, or it may arise because the spatial environment first influences students’ activities, environmental perceptions, or social interactions, which subsequently affect their stress levels (17). Nevertheless, most studies have relied on cross-sectional designs and simple linear models and have often interpreted statistical associations between spatial characteristics and restorative outcomes as environmental effects. This approach not only limits causal inference but may also contribute to inconsistencies across studies (7, 19, 47). Although a small number of studies have identified indirect pathways involving attitudinal responses and behavioral support, the available evidence remains insufficient to establish a robust explanatory model for university campus contexts (21, 36).

Research on urban environments and nature-health relationships has already proposed several restorative mechanisms with broad applicability. Environmental characteristics may facilitate attentional, stress-related, and affective restoration through perceived restorativeness, while also influencing health and well-being through physical activity, social connections, and reduced exposure to environmental stressors (71, 72, 74). The urban restoration model proposed by Bornioli and Subiza-Pérez (73) further suggests that restoration may arise from passive processes elicited by perceptual environmental characteristics or from active processes supported by activities, personal meanings, and place-based experiences (73). Accordingly, this review does not assume that the underlying mechanisms of psychological restoration are inherently specific to university campuses. Rather, the contribution of campus-based research lies in testing whether these broadly applicable mechanisms extend to university students and in identifying how academic stress, routine patterns of space use, and campus environmental conditions moderate the relevant pathways.

Future research should focus on testing the relative contributions and boundary conditions of existing restorative mechanisms in university campus settings rather than merely replicating direct associations between spatial characteristics and restorative outcomes. Specifically, studies should distinguish the direct effects of spatial characteristics on attentional, stress-related, and affective restoration from indirect effects operating through perceived restorativeness, environmental attitudes, activity participation, and social interaction. Potential moderators—including baseline stress or attentional fatigue, the purpose of space use, the frequency and duration of exposure, stage of study, and social context—should also be examined. Longitudinal, quasi-experimental, repeated-measures, and mediation designs could help establish the temporal ordering of environmental exposure, restorative processes, and broader health outcomes while reducing the overinterpretation of cross-sectional associations as causal effects. The resulting contribution should therefore be framed as a context-specific test and extension of broadly applicable restoration theories rather than as the development of an entirely independent model of restoration for university campuses.

### 4.4 Greater quantification of environmental characteristics and standardization of restoration outcome measurement are needed

To assess environmental characteristics and exposures, most previous studies have measured the characteristics and constituent elements of university common spaces using conventional interviews and questionnaire surveys. These approaches are time-consuming and make it difficult to convert qualitative spatial elements into quantitative indicators, limiting cross-campus comparisons. For example, without standardized methods for measuring vegetation cover, green view ratio, sky visibility, building enclosure, interface complexity, facility distribution, and accessibility, results are difficult to compare across studies and translate into actionable design thresholds and optimization strategies (18, 26, 32, 66). In recent years, street-view imagery, deep learning, GIS analysis, parametric modeling, and multimodal data collection have created new opportunities for objectively identifying campus spatial elements, but these technologies must be combined with clear theoretical hypotheses and reliable measures of psychological restoration (75–78).

To assess restoration outcomes, studies commonly use subjective questionnaire measures of students’ restorative experiences or physiological experiments. Questionnaire studies generally use validated or self-developed scales, including the Perceived Restorativeness Scale (PRS), Restoration Outcome Scale (ROS), and WHO-5 Well-Being Index (16, 33, 39). Physiological experiments mainly use indirect measures such as heart rate, blood pressure, and electrodermal activity (6, 9). Subjective questionnaire ratings can reflect university students’ perceptions and experiences at the micro-environmental level, while physiological measures can help address the limitations of self-report; however, physiological results can be influenced by the experimental context, individual differences, and external interference (79, 80). Scales, as common tools for subjective evaluation, can systematically assess participants’ overall responses, helping to clarify the structure of restoration outcomes and advance empirical research (79). Overall, future research should establish an integrated methodological framework in which objective spatial measurement, subjective perceptual evaluation, and psychological and behavioral restoration outcomes can be used to corroborate one another, thereby improving the stability and explanatory power of the evidence.

### 4.5 Limitations

This study has several limitations. First, the search was conducted in a limited set of databases and sources spanning multidisciplinary, health, environmental, psychological, and Chinese-language literature; however, PsycINFO and Scopus were not searched. Consequently, some studies published in psychology, architecture, landscape architecture, or urban studies journals, as well as studies not indexed in the searched databases or published in other languages, may have been missed. In addition, the use of specific phrases referring to university common spaces may have limited the retrieval of otherwise eligible studies described using alternative terms, such as “campus green space” or “campus landscape.” The gaps identified in this review should therefore be interpreted as patterns within the retrieved and included evidence rather than as an exhaustive representation of the entire field. Second, the included studies differed substantially in spatial type, sample characteristics, study design, and measurement tools. We therefore used a narrative synthesis rather than a meta-analysis and could not estimate specific effect sizes or compare the strength of effects across spatial elements. Third, the purpose of a scoping review is to map the evidence and identify knowledge gaps rather than to make strict causal inferences across all included studies. Future reviews could combine systematic-review methods, quality appraisal, or meta-analysis to provide a more detailed synthesis of evidence on specific spatial types, restoration indicators, or pathways of influence.

## 5 Conclusions and future directions

### 5.1 Main conclusions

This review systematically synthesized the current evidence on the relationship between university common spaces and university students’ psychological restoration. The evidence indicates the following: (1) the characteristics and constituent elements of university common spaces are consistently and positively associated with students’ restoration; (2) the main restoration indicators are attention restoration, stress reduction, regulation of negative emotions, vitality restoration, and reduced loneliness, all of which are influenced to varying degrees by common-space characteristics and their constituent elements; (3) common-space characteristics and constituent elements may act directly on psychological restoration and indirectly through attitudinal and behavioral-support factors, including perceived restorativeness, environmental satisfaction, spatial preference, place attachment, physical activity, and social interaction. Overall, this scoping review provides an evidence base for understanding the restorative potential of university common spaces and highlights limitations in spatial coverage, quantification of constituent elements, integration of restoration indicators, and mechanism identification.

### 5.2 Future research directions

Given the distribution of existing evidence and the limitations of prior research, future studies can advance research in five areas: environmental characteristics, restoration outcomes, research direction, methodology, and application.

#### 5.2.1 Environmental characteristics: consider common-space types together and examine their constituent elements systematically

Restorative-environment design should consider the synergistic effects of natural and built environmental elements; well-designed built environments can also have positive restorative effects. Future research should not be limited to campus vegetated spaces and waterfront spaces but should include plazas, courtyards, sports fields, and other university common spaces in comparative frameworks, while systematically examining the joint effects of architectural, landscape, and facility-support elements. A classification system covering space types, constituent elements, and use contexts will enable more accurate identification of the restorative characteristics of different university common spaces.

#### 5.2.2 Restoration outcomes: use integrated indicators to assess university students’ psychological restoration

Future research should treat university students’ psychological restoration as a multidimensional process rather than a change in a single emotion or stress level. Based on Attention Restoration Theory and Stress Reduction Theory, the five indicators of attention restoration, stress reduction, regulation of negative emotions, vitality restoration, and reduced loneliness can be combined into a measurement framework with greater explanatory power. Reports should also specify scale sources, adaptation procedures, reliability and validity testing, and applicable contexts to improve comparability across studies.

#### 5.2.3 Study direction: strengthen research on the processes and effects of psychological restoration among students in university common spaces

Future research should move beyond asking whether university common spaces are effective and examine how they work, thereby providing a more comprehensive understanding of the processes through which common spaces influence students’ psychological restoration and the resulting restorative effects. Study designs should make greater use of longitudinal follow-up, natural experiments, quasi-experiments, and structural equation modeling, with particular attention to mediation pathways through which common-space characteristics influence restoration via attitudes and behavioral support. Differences in the roles of perceived restorativeness, environmental satisfaction, spatial preference, place attachment, physical activity, and social interaction across space types and student groups should be examined.

#### 5.2.4 Study methods: combine emerging technologies with existing quantitative methods

To measure the constituent elements of university common spaces, future research can supplement conventional questionnaires, interviews, and field observations with street-view image recognition, deep learning, GIS analysis, parametric measurement, and multimodal perceptual data. These methods can improve the objectivity and reproducibility of spatial-element identification and facilitate standardized large-scale data collection. At the same time, emerging technologies should serve clearly defined theoretical and design questions and should be combined with validated restoration scales, behavioral observations, and physiological indicators as needed to establish a research approach that allows findings from multiple sources to be cross-validated.

#### 5.2.5 Study applications: improve the research paradigm to guide improvements to the design of university common spaces for psychological restoration

To support the development of healthy campuses, future research should further translate evidence into design practice. Restorative design strategies for different common-space types can be developed around four steps: clarifying current problems in university common spaces, identifying key restorative factors, identifying pathways and effects, and developing strategies for design optimization. Translating research findings into actionable spatial indicators, recommendations for facility configuration, and guidance on space use can help university common spaces better support students’ mental health and everyday restoration needs.

## Data Availability

The original contributions presented in the study are included in the article, further inquiries can be directed to the corresponding author.
